# Ipsilesional Mu Rhythm Desynchronization and Changes in Motor Behavior Following Post Stroke BCI Intervention for Motor Rehabilitation

**DOI:** 10.3389/fnins.2019.00053

**Published:** 2019-03-06

**Authors:** Alexander B. Remsik, Leroy Williams, Klevest Gjini, Keith Dodd, Jaclyn Thoma, Tyler Jacobson, Matt Walczak, Matthew McMillan, Shruti Rajan, Brittany M. Young, Zack Nigogosyan, Hemali Advani, Rosaleena Mohanty, Neelima Tellapragada, Janerra Allen, Mohsen Mazrooyisebdani, Leo M. Walton, Peter L. E. van Kan, Theresa J. Kang, Justin A. Sattin, Veena A. Nair, Dorothy Farrar Edwards, Justin C. Williams, Vivek Prabhakaran

**Affiliations:** ^1^Department of Radiology, University of Wisconsin–Madison, Madison, WI, United States; ^2^Department of Kinesiology, University of Wisconsin–Madison, Madison, WI, United States; ^3^Institute for Clinical and Translational Research, University of Wisconsin–Madison, Madison, WI, United States; ^4^Department of Educational Psychology, University of Wisconsin–Madison, Madison, WI, United States; ^5^Center for Women’s Health Research, University of Wisconsin–Madison, Madison, WI, United States; ^6^Department of Neurology, University of Wisconsin–Madison, Madison, WI, United States; ^7^Department of Biomedical Engineering, University of Wisconsin–Madison, Madison, WI, United States; ^8^Neuroscience Training Program, University of Wisconsin School of Medicine and Public Health, Madison, WI, United States; ^9^Department of Psychology, University of Wisconsin–Madison, Madison, WI, United States; ^10^Clinical Neuroengineering Training Program, University of Wisconsin–Madison, Madison, WI, United States; ^11^Medical Scientist Training Program, University of Wisconsin School of Medicine and Public Health, Madison, WI, United States; ^12^Department of Electrical and Computer Engineering, University of Wisconsin–Madison, Madison, WI, United States; ^13^Department of Materials Science and Engineering, University of Wisconsin–Madison, Madison, WI, United States; ^14^Department of Neurological Surgery, University of Wisconsin–Madison, Madison, WI, United States; ^15^Department of Psychiatry, University of Wisconsin–Madison, Madison, WI, United States

**Keywords:** brain–computer interface;, hemiparesis; r-squared; coherence; chronic; acute; neuroplasticity; homunculus

## Abstract

Loss of motor function is a common deficit following stroke insult and often manifests as persistent upper extremity (UE) disability which can affect a survivor’s ability to participate in activities of daily living. Recent research suggests the use of brain–computer interface (BCI) devices might improve UE function in stroke survivors at various times since stroke. This randomized crossover-controlled trial examines whether intervention with this BCI device design attenuates the effects of hemiparesis, encourages reorganization of motor related brain signals (EEG measured sensorimotor rhythm desynchronization), and improves movement, as measured by the Action Research Arm Test (ARAT). A sample of 21 stroke survivors, presenting with varied times since stroke and levels of UE impairment, received a maximum of 18–30 h of intervention with a novel electroencephalogram-based BCI-driven functional electrical stimulator (EEG-BCI-FES) device. Driven by spectral power recordings from contralateral EEG electrodes during cued attempted grasping of the hand, the user’s input to the EEG-BCI-FES device modulates horizontal movement of a virtual cursor and also facilitates concurrent stimulation of the impaired UE. Outcome measures of function and capacity were assessed at baseline, mid-therapy, and at completion of therapy while EEG was recorded only during intervention sessions. A significant increase in r-squared values [reflecting Mu rhythm (8–12 Hz) desynchronization as the result of attempted movements of the impaired hand] presented post-therapy compared to baseline. These findings suggest that intervention corresponds with greater desynchronization of Mu rhythm in the ipsilesional hemisphere during attempted movements of the impaired hand and this change is related to changes in behavior as a result of the intervention. BCI intervention may be an effective way of addressing the recovery of a stroke impaired UE and studying neuromechanical coupling with motor outputs.

**Clinical Trial Registration:** ClinicalTrials.gov, identifier NCT02098265.

## Introduction

### Stroke

Stroke is a leading cause of acquired adult long-term disability in the United States (Benjamin et al., 2017) and occurs when blood supply to the brain is compromised, leading to functional deficits that may affect activities of daily living (ADLs). Approximately 85% of patients who suffer and survive a new or recurrent stroke in the United States each year require rehabilitation (Yang et al., 2017). Six months post-stroke, nearly 50% of survivors have some residual motor deficits (Benjamin et al., 2017). By 2050, stroke burden on the United States economy will approach $2.2 trillion (Benjamin et al., 2017). Despite advances in acute stroke care, the estimated direct and indirect costs of stroke continue to escalate and are disproportionally associated with long-term care and rehabilitation (Benjamin et al., 2017). Current standard of care seems insufficiently developed to treat long-term motor deficits, potentially further burdening patients as untreated motor impairment can lead to deconditioning and underutilization of the affected upper extremity (UE), a consequence deemed, learned non-use (LNU) (Schaechter, 2004).

### Customary Care and the Opportunities for Improvement

Several rehabilitation techniques are traditionally used for stroke recovery including conventional physical-occupational-speech therapies, provided in acute care settings as well as newer motor therapies such as constraint-induced movement therapy (CIMT), robot-aided therapy, transcranial direct current stimulation (tDCS), transcranial magnetic stimulation (TMS), and virtual reality (VR) (Kollen et al., 2006; Lindenberg et al., 2010; Fleet et al., 2014; Young et al., 2014a,b,c; Laver et al., 2015; Song et al., 2015; Babaiasl et al., 2016; Smith and Stinear, 2016). Importantly, a much different level of evidence exists for CIMT and traditional therapies than experimental therapies such as tDCS and VR-based approaches. Existing pharmacological treatments, Botox injections for example, and traditional physical therapy methods primarily serve to treat symptoms associated with stroke (Benjamin et al., 2017) and may not focus on bringing about basic changes to the underlying impaired brain function associated with relevant post-stroke pathologies. Patients with UE motor impairment traditionally receive rehabilitation regimens that involve passive, repetitive movement of the impaired limb without directly linking brain activity to these movements (Dromerick et al., 2009). Whereas passive movement repetition can be an effective rehabilitation strategy, recovery can be slow, and suboptimal. In contrast, linking brain activity to movement is important for motor skill learning (e.g., walking, running, throwing, writing, etc.) and the formation of central to peripheral connections. Leveraging this innate and robust motor learning circuitry, harnessing brain plasticity (Thakor, 2013), may be the next step toward improve patient outcomes.

### Motor Recovery

Research suggests that motor recovery post-stroke, similar to motor learning, requires specific internal and external environmental conditions (Power et al., 2011; Wenger et al., 2017). For example, lesion load is a limiting factor as sufficient existing neural-architecture is needed for motor recovery to occur (Power et al., 2011). Recovery likely manifests either by the return of function to surviving neural architecture, or via neural reorganization and neural network remapping of proximal (i.e., near-by) neural architecture (Gazzaniga, 2005; Jones, 2017). Perhaps such processes may even be related. If neuroplasticity in the motor system, though likely attenuated by age, is continuous (Gazzaniga, 2005) over the life course (Power et al., 2011; Wenger et al., 2017), long-studied learning theories such as Hebbian plasticity and classical conditioning might be better integrated in treatment designs to aid recovery of stroke impaired UE motor capacities (Power et al., 2011; Remsik et al., 2016). The incorporation of neurorehabilitation techniques has yielded operational clinical therapies and devices (Pfurtscheller et al., 1997, 2005; Neuper and Pfurtscheller, 2001; Pineda, 2005; Felton et al., 2007; Schalk et al., 2008; Power et al., 2011; Kuiken et al., 2013; Young et al., 2014a; Wenger et al., 2017). As a number of existing approaches suffer from issues of high cost, passive movement repetition, large equipment, personnel and time constraints it is crucial efforts are made to pursue more expedient and efficacious means of rehabilitation, improve our quality of care, and better serve our survivors.

### Sensorimotor Rhythms

Human brain rhythms associated with motor output, sensorimotor rhythms (SMRs), are recorded superficial to the motor and somatosensory cortical strip of the brain (electrode sites C3 and C4) and originate according to homuncular organization (Pfurtscheller et al., 1997; Birbaumer et al., 2006). At the motor cortical strip (generally, Brodmann areas 3–6), each brain hemisphere desynchronizes with imagined, attempted, and also preparation of movement. This phenomenon is known as event-related desynchronization (ERD). Specific frequency bands have been associated with specific aspects of event-related motor behaviors (Pfurtscheller et al., 1997, 2005; Felton et al., 2007; Schalk et al., 2008; Song et al., 2014; Young et al., 2014b). In normal effortful movement, Mu rhythms of the contralateral cortex are desynchronized and attenuated (ERD) as movements are planned and executed (Pfurtscheller et al., 1997). This is followed by an increased presence of Beta rhythm ERD in the contralateral motor cortex which is associated with the later stages of motor command output and control (Pineda, 2005). After the completion, or at the cessation of movement, the SMRs in Mu and Beta frequency bands synchronize (ERS). ERD and ERS were key elements in the development and use of early BCIs for the rehabilitation of motor functions (Pfurtscheller et al., 1997, 2005; Nam et al., 2011). The early designs confirmed that ERD or ERS in specific spatial areas and neural networks (e.g., thalamocortical networks, frontoparietal networks) associated with a task or triggered events can be utilized to control a device or output command (Pfurtscheller et al., 1997; Neuper and Pfurtscheller, 2001).

Mu and Beta sensorimotor rhythms (SMRs) in human subjects are recorded exclusively over sensorimotor areas at frequencies of about 10–20 Hz (Pfurtscheller et al., 1997; Birbaumer et al., 2006). Two basic strategies in SMR-based control have been introduced for motor rehabilitation in stroke patients: motor imagery (Wolpaw et al., 1991; Ortner et al., 2012; Irimia et al., 2016) and attempted movement-based approaches (Wolpaw et al., 1991; Schalk et al., 2004; Young et al., 2014a,b,c). Either approach utilizes essentially overlapping neural architecture to provide input signals (electrophysiological recordings by the EEG cap) to the BCI. The authors of this study designed the protocol to utilize attempted hand movements during the intervention according to the logic that a motor therapy intended to restore volitional motor function of the affected UE should utilize voluntary attempted movements of that impaired hand in a continuous effort to improve the participant’s UE capacity and performance.

### Brain–Computer Interface (BCI) and Electroencephalography for Assistive Design

Noninvasive brain–computer interfaces (BCIs), which utilize ancillary adjuvant peripheral devices and electrical muscle stimulation, as well as invasive BCI approaches with electrodes implanted in the skull, have been introduced (Wolpaw et al., 1991; Leuthardt et al., 2004; Schalk et al., 2004, 2008; McFarland et al., 2006; Felton et al., 2007) as contemporary intervention and rehabilitation techniques following neural disease or trauma, such as stroke. Devices similar to what was utilized in this research are controlled by input signals generated by scalp electroencephalographic (EEG) recordings from electrodes superficial to the sensorimotor cortices. EEG signals associated with various components of voluntary movement are identified and translated into a device command or specified output (Pfurtscheller et al., 1997, 2005; Felton et al., 2007; Schalk et al., 2008; Wilson et al., 2012), like activation of an FES pad (Song et al., 2014; Young et al., 2014a,b). BCIs can monitoring volitional modulation of electrical brain rhythms and execute an augmentative, facilitative, or rehabilitative command in the presence or absence of such signals.

### Adjuvants

In this study, EEG driven BCI was linked to tongue stimulation (TS) via a Tongue Display Unit (TDU) (Kaczmarek, 2011; Wilson et al., 2012) (designed as a visual supplementation for any participant with visual impairments) and FES, which can act not only as therapeutic adjuvants but, when tied to intent-to-move brain signals, also provide users with multi-modal feedback as a form of monitoring and reward for producing relevant brain activity patterns (SMR modulation) during tasks. Adjuvant stimulation may not only aid execution of the motor plan by causing the contraction of the impaired UE musculature but may also help the user learn new movement strategies for the impaired extremity. Adjuvant-induced proprioceptive and general afferent inputs to the motor system complete the BCI design’s replication of the native neurobiological closed-loop motor system. Such adjuvant-aided volitional movement may not only make a movement possible but also contribute ancillary components for motor learning. Rewards of tactile, kinesthetic feedback to the system and the visual revelation of a previously impaired appendage now voluntarily animated may prove powerful (Moe and Post, 1962; Krafi et al., 1992; Popovic et al., 2009; Howlett et al., 2015) reinforces.

### Evidence

Growing evidence from our lab (Young et al., 2014a,b,c; Song et al., 2015) and other groups (Hill et al., 2006; Daly and Wolpaw, 2008; Daly et al., 2009; Caria et al., 2011; Muralidharan et al., 2011; Ang and Guan, 2013; Varkuti et al., 2013; Bundy et al., 2017) suggest that noninvasive EEG-BCI-FES systems hold potential for facilitating recovery in the chronic phase after stroke by linking central nervous system (CNS) commands, or brain activity, with distal motor effectors (the manifestation of the motor plan via trained muscle synergies) of the peripheral nervous system (PNS). Integration of the aforementioned command with facilitated movements within strict reinforcement constraints (e.g., task accuracy: drop the cup, move the ball or not) might thereby better harness neuroplastic capacities leading to functional gains in recovery for individuals with stroke related hemiparesis. Previous studies suggest that change in the pattern of brain activity linked to attempted movements of the affected hand contributes to motor re-conditioning and induces brain plasticity or reorganization which, if properly directed and reinforced, should lead to improvement in a stereotyped motor function of interest (Daly et al., 2009; Caria et al., 2011; Muralidharan et al., 2011; Varkuti et al., 2013). This is of special importance for patients in the chronic phase (generally >6 months post stroke) of recovery who may have little to no residual function in the affected arm, in addition to learned compensatory motor strategies (Muralidharan et al., 2011). Given that these participants have also likely exhausted other forms of intervention available to them through standard healthcare channels, it is imperative to explore novel intervention technologies that show promise in this population.

### Overview of This Study

It was hypothesized that (1) the EEG-BCI-FES intervention sessions would result in increased hemispheric desynchronization levels of Mu (8–12 Hz) rhythm and, or Beta (18–26 Hz) band signals over the ipsilesional motor cortices, as reflected by increased r-squared values (i.e., lower power in the impaired hand movement trials compared to rest), and (2) changes in functional connectivity (coherence) are greatest in the affected contralateral (ipsilesional) motor cortex and, over time, are associated with beneficial behavior and quality of life improvements as measured by objective and subjective measures of upper extremity motor function and activities of daily living. This interim analysis, of the larger ongoing prospective randomized crossover-controlled clinical trial, seeks to determine whether greater desynchronization of motor related SMRs in the ipsilesional hemisphere during attempted movements of the impaired hand are related to changes in behavior as a result of intervention.

## Materials and Methods

### Subjects and Design

#### Ethics Statement

Participants were recruited from the greater Madison, WI, United States area as part of an on-going prospective randomized, cross-over controlled design stroke rehabilitation study investigating interventional BCI targeting UE motor function. This study is approved by the University of Wisconsin Health Sciences Institutional Review Board (Study ID 2015-0469); all subjects provided written informed consent upon enrollment. A CONSORT flow diagram is made available in Supplementary Figure S1.

#### Recruitment and Enrollment

This on-going study, registered with ClinicalTrials.gov (study ID^1^ NCT02098265), employs an open call for participants with a wide range of (1) UE hemiparesis resulting from stroke, (2) time-since-stroke, (3) stroke type, (4) lesion location, (5) number of previous strokes, (6) and stroke severity. Subsequent to informed, written consent, stroke survivors were randomized by permuted-block design accounting specifically for gender, stroke chronicity, as well as severity of motor impairment as measured by the Action Research Arm Test (ARAT) (Lang et al., 2008) (*n* = 21, mean age = 61.6 years ± 15.3 years, 12 female, 13 right lateralized lesion, mean chronicity = 1127 days ± 1326.5 days, median chronicity 588 days, 11 with severe UE motor deficit, mean baseline ARAT score of impaired side = 26.6 ± 26.1). Chronicity is measured as time since stroke, in days, to baseline measurement day. Participant characteristics are displayed in Table 1. This interim analysis of the larger ongoing study seeks to elucidate the electrophysiological consequences and associations of BCI participation and the authors focus specifically on the behavioral (primary outcome) associations in another manuscript published in tandem with this effort (Remsik et al., 2018).

**Table 1:** Participant characteristic and ARAT score.

Participants	Age (years)	Chronicity (days)	Severity	Clinical cause	Baseline	Completion	Follow-up	ARAT
				Lesion location	ARAT	ARAT	ARAT	change
1	47–51	160	Severe	L-Lateral medulla	3	2	7	–1 (4)
2	49–53	490	Severe	R-MCA stroke	3	4	8	^∗^1 (5)
3	76–80	658	Mild	Leg/periventricular white, MHR	57	57	57	0 (0)
4	67–51	2723	Severe	R-PLIC putamen	23	40	39	^∗^17 (16)
5	81–85	580	Mild	Cerebellar vermis	47	52	52	^∗^5 (5)
6	73–77	197	Severe	R-Prefrontal, midfrontal, temporal	0	0	3	0 (3)
7	62–66	101	Mild	R-White matter	56	57	57	^∗^1 (1)
8	40–44	2645	Severe	R-Frontal parietal	7	7	7	0 (0)
9	55–59	588	Severe	R-MCA	3	4	0	^∗^1 (-3)
10	45–49	452	Severe	L-Hemorrhagic stroke	0	2	0	^∗^2 (0)
11	30–34	494	Mild	L-ICA	57	57	57	0 (0)
12	60–64	44	Mild	L-PCA	57	57	57	0 (0)
13	57–61	849	Mild	L-MCA	57	57	57	0 (0)
14	44–48	3017	Severe	R-MCA/R-FI	3	4	5	^∗^1 (2)
15	69–73	790	Severe	R-MCA/R-TP	3	0	3	–3 (0)
16	78–82	631	Mild	R-Occipital	57	57	57	0 (0)
17	75–79	5125	Severe	R-MCA/ACA	9	11	10	^∗^2 (1)
18	42–46	177	Mild	L-MCA	57	57	57	0 (0)
19	62–66	392	Severe	R-Frontal hematoma	3	5	16	^∗^2 (13)
20	55–59	2767	Mild	R-VAOA, subarachnoid hemorrhage	57	57	57	0 (0)
21	69–73	783	Severe	R-MCA	0	0	0	0 (0)
(A) Mean	61.6	1127			26.6	27.9	28.9	1.3 (2.2)
Median	61.9	588			9	11	16	0 (0)
SD	15	1327			26.4	26.6	25.9	3.9 (4.5)
(B) Mean	61.1	1289			11.4	13.4	14.8	2 (3.4)
Median	64	584			3	4	7	1 (1.5)
SD	13.5	1497			18	20.2	19.6	4.7 (5.2)

*ARAT, Action Research Arm Test; MCA, middle cerebral artery; ICA, internal carotid artery; PCA, posterior cerebral artery; FI, frontoparietal infarct; TP, temporal frontal-parietal; ACA, anterior cerebral artery; MHR, motor hand region; VAOA, vertebral artery origin aneurysm; L, left; R, right; ARAT change, completion-baseline (follow-up baseline). (A) Indicates descriptive statistics for all (*n* = 21) participants; (B) indicates descriptive statistics for (*n* = 14) participants able to achieve ARAT improvements (ceilings removed). Positive change in outcome measure at competition of BCI intervention denoted by ‘^∗^’.*

#### Inclusion–Exclusion Criteria

**FIGURE 1:**
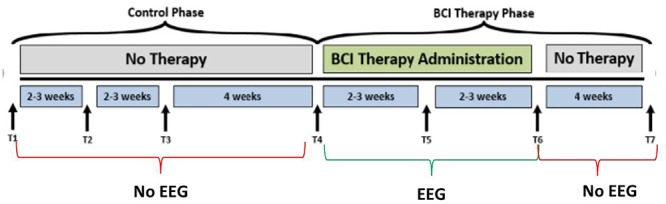
Study design. The time-points at which neuroimaging data were collected are represented by, T1, control baseline; T2, control middle; T3, control completion; T4, intervention baseline; T5, mid-intervention; T6, completion of intervention; T7, 1-month post-intervention. While the crossover control group (DTG) completed visits T1 through T7, the immediate therapy (ITG) group completed only visits T4 through T7. EEG-BCI-FES intervention is only administered during the BCI Therapy Phase (green), from baseline (T4) to completion (T6), and EEG recordings are neither acquired between T1 and T4, nor between T6 and T7 during which only MRI and behavioral testing batteries are administered. EEG data were only collected during the intervention phase.

Participants age 18 years or older with persistent UE motor impairment resulting from stroke and no other known neurologic (cognitive, expressive), psychiatric (affect), or developmental disabilities were included. Exclusion criteria were: allergy to electrode gel, surgical tape, metals, concurrent treatment for infectious disease, apparent lesions or inflammation of the oral cavity, pregnancy or intention to become pregnant during the course of the study, or any contraindication for magnetic resonance imaging (MRI). Subjects from the greater study cohort were excluded from the presented analyses if they (1) failed to complete at least 9 of 15, two-hour BCI intervention sessions occurring at least twice each week, (2) failed to complete all four MRI and behavioral testing sessions occurring in the intervention phase (Figure 1).

#### Randomization and Study Schema

Participants, when assigned to the intervention phase, have at least 9 and up to 15 EEG-BCI-FES intervention sessions (two-to-three sessions/week) wherein they receive EEG-BCI-FES intervention lasting up to 2 h for a potential total dosing of 30 h of EEG-BCI-FES intervention. Along with the EEG-BCI-FES intervention sessions, subjects also receive fMRI and behavioral testing at four time points: prior to the first EEG-BCI-FES intervention session (baseline), after the first few weeks of intervention (midpoint), following the final intervention session (endpoint), and again 1 month after the endpoint assessment (follow-up). Subjects assigned to the delayed intervention group (DTG) are encouraged to continue with their normal and customary care while in the delay period. While in the delay period, participant EEG data are not recorded and participants are instructed not to use a BCI device. During this time, there are three assessment visits consisting of MRI and behavioral testing which are matched in sequence and duration to those conducted in the BCI intervention period as demonstrated in Figure 1. After completion of the delay period, these participants cross over into the intervention phase and are assessed in accordance with previously described methods. All data and time points analyzed and presented herein were recorded during the BCI intervention period only, for all participants. EEG data were only collected during the intervention phase.

### The BCI System

The BCI system and intervention sequence were consistent with those previously described (Wilson et al., 2012; Song et al., 2014, 2015; Young et al., 2014a,b,c) using BCI2000 software (Schalk et al., 2004) version 2 with in-house modifications for input from a 16-channel EEG cap and amplifier (Guger Technologies) and integration with the ball and target gaming visual display as well as tongue stimulation (TDU 01.30 Wicab Inc.) (Kaczmarek, 2011) and functional electrical stimulation (FES) (LG-7500, LGMedSupply; Arduino 1.0.4). FES of the UE was delivered through a pair of 2” × 2” square electrodes, commercially available stimulus isolator units, which ensure clean, opto-electrical isolation, placed securely on the affected forearm using highly conductive Electrolyte Spray and produced by the LG-7500 Digital Muscle Stimulator LGMedSupply, Cherry Hill, NJ, United States). The electrodes were placed to facilitate either a grasping motion (finger flexion), or finger extension according to participant preference. Specific placement sites were superficial to flexor digitorum superficialis to facilitate hand and finger flexion, or superficial to extensor digitorum communis to facilitate hand and finger extension. The natural absence of a flexor digitorum superficialis tendon to the fifth digit in some individuals was not considered in this study design. Stimulation was controlled through the PC using an Arduino Uno R3 (Adafruit Industries, New York, NY, United States) and a simple Reed-Relay circuit, with the amplitude set to elicit observable muscle activation (e.g., finger grasping) without pain. The pulse rate of the stimulation was set to 60 Hz in order to produce tetanic contraction of the muscle and the pulse width was set to 150 μs. The input signal, initially set to zero, was adjusted by steps of 0.5 mA, unless the stimulation became uncomfortable for the subject. The device was never set to deliver an output greater than 5 mA. In previous publications, the TDU (Kaczmarek, 2011) has been described and its use in a BCI paradigm detailed (Wilson et al., 2012). This BCI system uses the same TDU stimulation parameters as were reported previously (Wilson et al., 2012).

### Brief Overview of the Procedure (EEG Tasks)

#### EEG-BCI-FES Intervention

**FIGURE 2:**
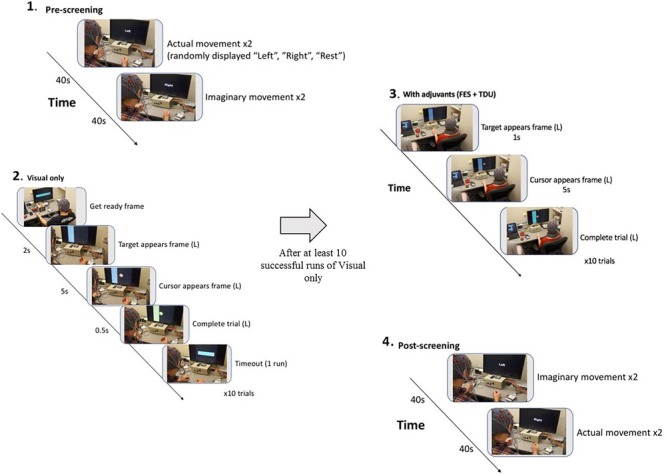
BCI intervention block design. **(1)** Pre-screening (two actual movement trials, two imagined movement trials). **(2)** Cursor task (≥10 trials with visual-only feedback). **(3)** Cursor task with adjuvant stimuli (≥10 trials with adjuvant stimuli). **(4)** Post-screening (two imagined movement trials, two actual movement trials).

Subjects went through intervention sessions on separate days. The number of EEG-BCI-FES intervention sessions varied across subjects with a mean of 13.8 ± 1.3. Each EEG-BCI-FES intervention session consisted of multiple runs of the ‘Cursor Task’ (mean of 31.3 ± 10.5 runs per session), about 1/3rd of which included only visual feedback, and roughly two thirds of which were comprised of BCI facilitated functional electric stimulation of the impaired hand and lingual electrotactile stimulation through a tongue display unit (TDU) (Kaczmarek, 2011; Wilson et al., 2012) (Figure 2). The EEG-BCI-FES device was driven by spectral power recordings from contralateral (to the hand active in the grasping task) EEG electrodes during cued attempted grasping movements of the hand which was designed to modulate the horizontal movement of a cursor (Schalk et al., 2008) in a computer display space as well as facilitate concurrent functional electrical stimulation of the participant’s impaired UE (should the target appear on the side corresponding to their stroke-impaired hand). BCI classifier inputs were therefore at C3 and C4, respectively in Mu (8–12Hz) and Beta (18–26Hz) frequency bands in this design. Each EEG-BCI-FES (closed-loop) intervention session was preceded by an open-loop pre-therapy screening phase and followed by an open-loop post-therapy screening phase (Figure 2). The successive order of intervention procedure was as follows: visual only, visual + FES, visual + FES + tongue feedback. All intervention sessions included in this analysis contained a similar distribution of these conditions across all participants.

#### Familiarization With BCI Device and Procedures

**FIGURE 3:**
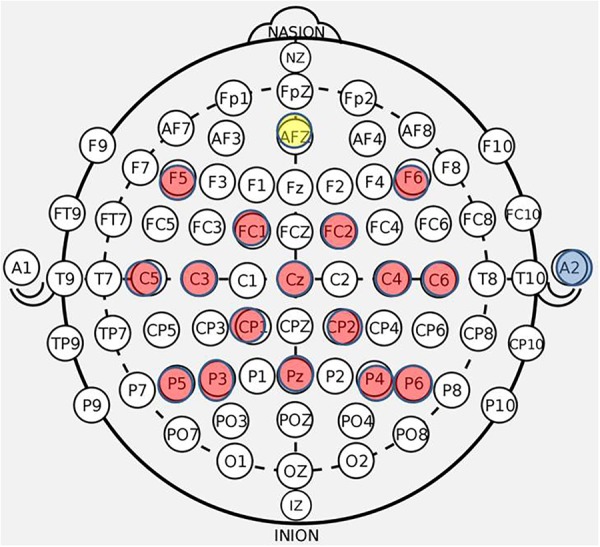
BCI cap array. Electrode array and cap arrangement for all *n* = 21 participants. Control signals generated at C3 and C4 electrodes for right and left hand movement trials, respectively. Ear clip always placed on the right ear.

The first BCI session was focused on assisting the participant to comprehend and engage the BCI device and protocol. Stroke survivors often present with a myriad of cognitive, affective, and physical impairments (Nair et al., 2015) and out of respect for individual participant needs and abilities, the researchers intended to provide at outset an opportunity for a generous orientation rather than rigorous acquisition. During this preliminary session, the EEG cap (Figure 3), FES device, and TDU device were faithfully administered as described previously (Wilson et al., 2012). Participants were verbally instructed before each session, and as needed, to aim for successful completion of BCI tasks and for each attempted movement to be performed to the participant’s autonomously elected level of comfort and ability. There were no participants in this study whom were unable to comprehend or participate successfully in the intervention protocol as a result of any associated cognitive or aphasic impairments associated with their stroke. The study design requires at least 10 runs for each closed-loop condition, per session; however, enforcement discretion was encouraged until a participant demonstrated task comprehension during the first BCI intervention session.

### Description of the Raw EEG Data

EEG data were recorded using a 16-ch bioamplifier (g.USBamp; G.TEC Medical Engineering GmbH, Austria) from 16 active electrodes using a g.GAMMA cap (F5, C5, FC1, C3, P5, F6, C6, P6, Pz, P4, P3, FC2, Cz, CP2, C4, CP1) (Figure 3) according to 10-20 EEG electrode placement system with a right ear-lobe reference in a BCI2000 system environment (Schalk et al., 2004). The frequency bandwidth of the recorded signals was 0.1–100 Hz, with an additional notch-filter applied at 60 Hz. The sampling rate was 256 Hz. During each of the screening phases (pre- and post-therapy) EEG data were collected in four separate runs. Each screening EEG data file contained 15 trials for rest, left hand and right hand movements (i.e., five trials for each of the three conditions), separated by an interstimulus interval of 1.5–2 s. The order of trials in a run was random. Each of the trials had a duration of 4 s. The first two runs of the pre-therapy screening phase and the last two runs of the post-therapy screening phase incorporated cued “attempted” hand movements. The last two runs of the pre-therapy screening phase and the first two runs of the post-therapy screening phase incorporated cued “imaginary” hand movements.

### Description of the EEG Data Analysis

The raw EEG data files were loaded into Fieldtrip (a MATLAB-based toolbox for advanced neurophysiological data analyses), and fully processed using tools incorporated in this toolbox (Oostenveld et al., 2011) and MATLAB environment^2^. The main processing steps for the EEG data collected during screening phases were as follows:

(1) Digital filtering with a high-pass filter cutoff of 4 Hz, and a low-pass filter cutoff of 30 Hz. (2) Extraction and grouping of trials according to condition (rest, left hand movement, right hand movement), movement type (attempted, imaginary), and the screening phase (pre, post). This resulted in 10 trials for each of condition/movement/screening phase combinations. (3) Identification (variance based: thresholds set to 10 and 250 μV^2^ for low and high variance signals, respectively) and repair of bad (noisy) channels (spline interpolation), followed by the removal of three trials showing the highest variance (Thomson, 1982; Mitra and Pesaran, 1999). The channel was identified as bad (noisy, poor connection, etc.) if the variance was <10 or >250 μV^2^ in more than three trials (Thomson, 1982; Mitra and Pesaran, 1999). The units of the variance were those of the data squared: as the EEG data units were in micro Volts, the variance units were squared micro Volts. If more than four channels were identified as bad, the data for that session were removed from further analysis (i.e., 20.4% of data were discarded by not meeting the defined criteria). At session level, this step resulted in 28 s of EEG data (7 trials × 4 s) for each condition/movement/screening phase combination set. (4) An average-reference montage was applied to the data (i.e., re-referencing from the original monopolar recordings). (5) Spectral analyses with Fourier transforms computed using a multi-taper method (Thomson, 1982; Mitra and Pesaran, 1999) at a 0.25 Hz resolution: this finally resulted in estimates of absolute spectral power sampled for every 1 Hz bin during the interval of 4–30 Hz, and cross-spectral density. The trial length was 4 s and the resolution of Fourier Transforms was 1/4 = 0.25 Hz. (6) Coherence estimation was calculated between all pairs of channels (120 pairs from 16 available scalp channels) at every 1 Hz frequency bin of the mentioned interval. Coherence was calculated as the absolute value of the ratio of the cross-spectrum to the square-root of the product of the two auto-spectra (as applied in Fieldtrip software). (7) Calculation of signed r-squared (r-squared: coefficient of determination) values from the absolute power estimates between left or right hand movements and rest trials, and between the two movement conditions (left vs. right). The r-squared values were signed in a such way that a large negative number (-) would mean larger “desynchronization” of the rhythm (Mu or Beta) (Pfurtscheller et al., 1997, 2005; Neuper and Pfurtscheller, 2001; Pineda, 2005). (8) Calculation of change (POST–PRE) in signed r-squared values: the following formula was used: -(POST–PRE), so one would obtain positive numbers for “increases” in desynchronization. This was done for easier interpretation of the associations of r-squared changes with behavior changes as the result of EEG-BCI-FES intervention. Here the “flipping” of values (in order to assess the “impaired hand,” L or R) was applied to the impaired R-hand scores to put them together with scores from the impaired L-hand subjects. (9) Calculation of the laterality index (LI) for averaged coherence values (i.e., average coherence of each site with all others), used to evaluate shifts in coherence, as: (C3 - C4)/(C3 + C4). (10) Change (POST–PRE) in coherence LI values: LI as a number becomes more positive if there is a shift toward Left, and more negative if there is a shift toward Right (as the result of intervention). Therefore, for POST–PRE change in LI, the impaired L-hand values were multiplied with (-1) and the impaired R-hand values remained unchanged, as they were originally calculated. This way, the “expected change” is positive and the associations with behavioral changes can be more seasily interpreted.

### Statistics

The independent variables were the signed r-squared values and the coherence estimates. At individual subject level, the data consisted of average estimates per each session for condition/movement/screening phase combination sets, and at group level the estimates consisted of grand averages over sessions of each individual subject data in the group (pre- and post-therapy scores averaged separately across sessions). Non-parametric statistical tests were run by calculating Monte-Carlo estimates of the significance probabilities and critical values from the permutation distribution (Maris and Oostenveld, 2007), followed by correction for multiple comparisons using false discovery rate (FDR) when no prior hypothesis was available. The *priori* hypotheses of expected changes in the r-squared values and coherence as the result of intervention time at C3 and C4 sites were tested using paired *t*-tests in MATLAB. Associations between the r-squared changes and the total number of intervention runs as well as behavioral changes (e.g., ARAT scores) were assessed using Pearson’s and Spearman’s correlation, respectively. Finally, the associations between the signed r-squared values with behavioral scores from several tests at baseline were assessed using Spearman’s or Pearson’s correlation coefficients, as appropriate. Thresholds for significance and trend toward significance were set *a priori* at *p* ≤ 0.05 and 0.05 < *p* < 0.1, respectively, for all statistical analyses.

### Description of the Behavioral Outcome Measures

The primary outcome measure was the ARAT. The ARAT is a 57-point test designed to assess specific changes in upper limb function with sub-components for grasp, grip, pinch, and gross motor movement (Hsieh et al., 1998). The secondary measures include: The Stroke-Impact Scale (SIS), widely used to measure quality of life in stroke survivors that consists of 8 dimensions and a composite disability score (Vellone et al., 2015). The SIS is a 59-item patient-reported outcome measure, covering eight domains; strength (4 items), hand function (5 items), mobility (9 items), ADLs (10 items), memory (7 items), communication (7 items), emotion (9 items), and handicap (8 items); the domains are scored on a metric of 0–100, with higher scores indicating better self-reported health (Vellone et al., 2015). The National Institutes of Health Stroke Scale (NIHSS) is a tool used by healthcare providers to objectively quantify impairments caused by a stroke (Ortiz and Sacco, 2008). The NIHSS is composed of 11 items, each of which scores a specific ability between zero and four with higher scores indicating increased impairment (Ortiz and Sacco, 2008). The Barthel scale, or Barthel ADL index, is a scale used to measure performance in ADLs (Shah et al., 1989). It utilizes ten variables describing ADL and mobility. The ten variables addressed in the Barthel scale are: presence or absence of fecal incontinence, presence or absence of urinary incontinence, help needed with grooming, help needed with toilet use, help needed with feeding, help needed with transfers (e.g., from chair to bed), help needed with walking, help needed with dressing, help needed with climbing stairs, help needed with bathing. This scale yields a score of 0–100 with higher scores indicating improved performance (Shah et al., 1989). Gross grasp grip strength was measured using a dynamometer (Nam et al., 2011). The Nine-Hole Peg Test (9-HPT) is a brief, standardized, quantitative test of UE function (Mathiowetz et al., 1985). The score for the 9-HPT is an average of the two trials (Mathiowetz et al., 1985). Mini-Mental State Examination (MMSE) is scored out of 30 (Pangman et al., 2000). An MMSE score of 27–30 is generally associated with normal memory: a score 10–26 could indicate mild to moderate dementia, and a score less than 10 suggests severe dementia (Pangman et al., 2000). The Center for Epidemiologic Studies-Depression (CES-D) scale is one of the most frequently used self-report measures of depressive experiences (Shinar et al., 1986). The CES-D contains 20 items that are summed so that scores have a potential range from 0 to 60, with higher scores indicating greater frequency of depressive experiences (Shinar et al., 1986).

### Analyses of Outcome Measures

Primary analysis was a paired-sample *t*-test to evaluate the statistical significance of ARAT and secondary outcome measure changes (i.e., SIS, NIHSS, Barthel scale, grip strength, 9-HPT, MMSE, and CES-D) between baseline, completion, and follow-up scores (Table 2).

**Table 2:** Summary of mean outcome measure scores for baseline, completion, and follow-up of the BCI training conditions.

Outcome measures	Baseline score	Completion score	Follow-up score	Change score	*p-*Value
Stroke Impact Scale (SIS) (Max = 100) SIS_Hand function_	33.6 (15) 38.1	39 (25) 37.5	39.8 (25) 39.7	5.4 (62)	0.482 ^∗^(0.050)
SIS_recovery_	50.1 (50) 23.7	53.4 (60) 24.9	54.6 (60) 21.8	3.3 (4.5)	0.509 (0.216)
**NIH Stroke Scale/Score (NIHSS)** (Max = 4)	3.8 (3) 3.5	3.8 (2.5) 3.1	3.7 (2.5) 3.1	0 (-0.1)	1.0 (0.749)
**Barthel Index-Total** (Max = 100)	91.4 (100) 14	92 (97) 13.9	92.8 (100) 14.8	0.6 (1.3)	0.431 (0.167)
**Grip strength** (lbs)	18.8 (8.3) 21.5	22.6 (14.3) 23.5	20.5 (5) 24.6	3.8 (1.7)	^∗^0.046 (0.246)
**9-HPT** (seconds)	17.7 (0) 22.8	15 (0) 19.1	14.4 (0) 20.3	–2.5 (-3.2)	0.083 (0.054)
**MMSE** (Max = 30)	27.2 (29) 3.8	27.8 (29) 2.7	28.3 (29) 2.7	0.6 (1)	0.467 (0.494)
**CES-D** (Max = 60)	7.6 (7.5) 5.8	7.8 (3) 9.9	5.6 (3) 5.9	0.2 (-2)	0.802 (0.096)
**Action Research Arm Test (ARAT)** ARAT_Total_ (Max = 57)	16.9 (9) 23	18.3 (11) 23.4	21.4 (16) 23.4	1.3 (4.3)	^∗^0.046 ^∗^(0.020)
ARAT_Grasp_ (Max = 18)	22 (3) 5.1	2.9 (5) 5.3	3.6 (6) 6.3	0.7 (01.5)	0.106 (0.163)
ARAT_Grip_ (Max = 12)	2.9 (2) 4.7	2.9 (3) 4.8	3.8 (4) 4.6	0.1 (0.9)	0.582 ^∗^(0.025)
ARAT_Pinch_ (Max = 18)	4.5 (1) 7.3	4.9. (0) 7.9	5.1 (4) 7.7	0.4 (0.6)	0.289 (0.106)
ARAT_Gross_ (Max = 9)	3.4 (5) 2.7	3.4 (6) 3	3.6 (6) 3	0 (0.3)	1.000 (0.453)

*Measures are reported as mean (median) ± SD. Change score and *p-*value are reported as mean scores change between baseline and completion (mean scores change between baseline and follow-pp). ARAT scores are reported as mean scores change with ceilings removed. ^∗^*p* < 0.05, ^∗∗^*p* < 0.01, ^∗∗∗^*p* < 0.001.*

## Results

### Results of Outcome Measures

Of the 21 participants who completed the study and met the aforementioned criteria, 14 participants had room for improvement in the primary outcome measure, ARAT (ARAT_total_), of which nine (64%) realized improved scores after intervention, both at immediate completion and 1 month after completion. Participant characteristics are summarized in Table 1 and group outcome measures are further described in Table 2. All participant assessments at each time point were averaged to give a metric of cohort motor function change at the group level. Secondary measures were similarly group averaged to determine cohort measure changes as a result of time in intervention as well as at the 1 month follow-up (Table 2). The primary analysis showed significant change in baseline scores and completion scores (Figure 1: T4, T6) in the primary outcome measure (ARAT) (*p* = 0.046), and change at follow-up (*p* = 0.020) (Figure 1: T7), change in Grip Strength was found to be significant by completion of intervention (*p* = 0.046). This particular finding did not persist at the 1-month follow-up time point. Statistical significance was observed in the baseline to follow-up score analyses (Figure 1: T4 to T7) not only for the primary outcome measure but also in secondary outcome measures of hand function (i.e., SIS Hand Function *p* = 0.05) (Table 2). All statistically significant findings were observed in measures of hand function. Additionally, the secondary analyses presented no significant results.

#### EEG Measures

**FIGURE 4:**
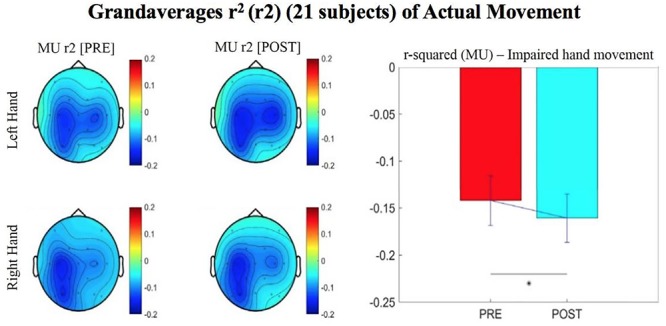
Topographical plots (topoplots) of grand averages for Mu rhythm (8–12 Hz) signed r-squared values at group level (*n* = 21). The bar plot shows the group means for the Mu rhythm signed r-squared values from the impaired hand attempted movement trials (vs. rest) at ipsilesional electrode site. Asterisk denotes statistical significance from a one-tailed paired *t*-test (*p* < 0.05). Error bars denote standard error of the mean. The majority of participants were left hand impaired. Prescreening, open-looped training (PRE) and open-looped post screening BCI training (POST) runs (color bar: [–0.2 = blue – 0.2 = red]). The majority of participants had a right lateralized lesion.

**FIGURE 5:**
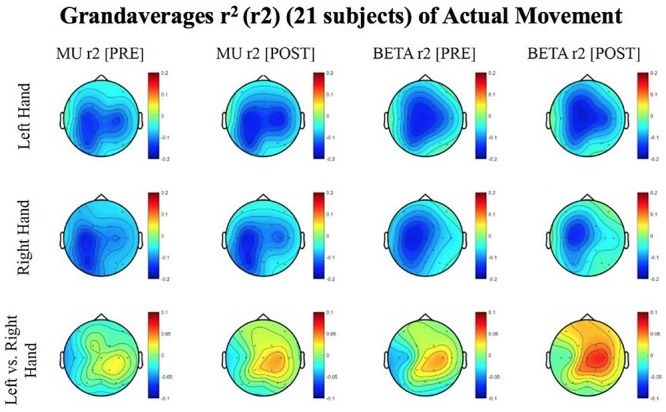
Topoplots of grand averages for signed r-squared values at group level (*n* = 21) for attempted movements. In the top two rows of topoplots, a larger negative value (blue) denotes stronger desynchronization (rest vs. left or right hand actual movement); in the bottom row of topoplots a larger positive value (red) denotes desynchronization (left vs. right hand actual movements). The mentioned distinction reflects the way in which the signed r-squared values were calculated in a rest vs. left/or right comparison, and in a left vs. right comparison. Prescreening, open-looped training (PRE) and open-looped post screening BCI training (POST) runs (color bar: [–0.2 = blue – 0.2 = red]). The majority of participants had a right lateralized lesion.

**FIGURE 6:**
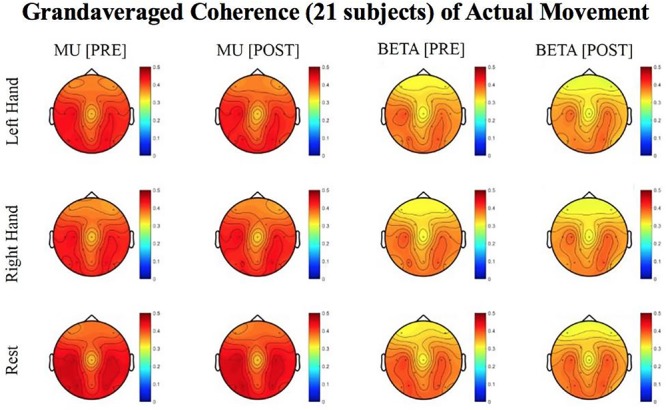
Topoplots of grand averaged coherence values at group level (*n* = 21) for Mu (8–12 Hz) and Beta (18–26 Hz) bands during attempted movement trials. Prescreening, open-looped training (PRE) and open-looped post screening BCI training (POST) runs (color bar: [0 = blue – 0.5 = red]). The majority of participants had a right lateralized lesion.

**FIGURE 7:**
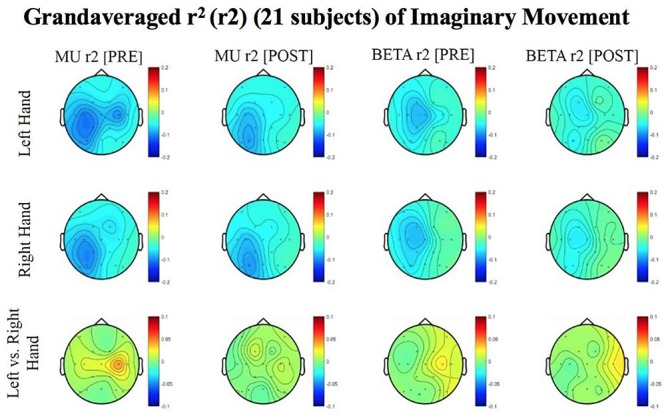
Topoplots of grand averages for signed r-squared values at group level (*n* = 21) for imaginary movements. Prescreening, open-looped training (PRE) and open-looped post screening BCI training (POST) runs (color bar: [0 = blue – 0.5 = red]). The majority of participants had a right lateralized lesion.

**FIGURE 8:**
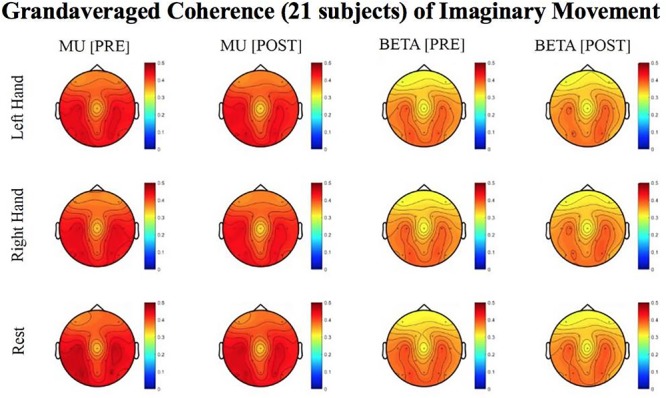
Topoplots of grand averaged coherence values at group level (*n* = 21) for Mu (8–12 Hz) and Beta (18–26 Hz) bands during imaginary movement trials. Prescreening, open-looped training (PRE) and open-looped post screening BCI training (POST) runs (color bar: [0 = blue – 0.5 = red]). The majority of participants had a right lateralized lesion.

Results reported below in Section “R-Squared” echoed in the graph in Figure 4, compared the signed r-squared values for the impaired hand separately from the non-impaired hand. The signed r-squared values from the Right-hand impaired participants at C3 (i.e., the ipsilesional motor site) were “pooled together” with the signed r-squared values from the Left-hand impaired participants at C4 (i.e., the ipsilesional motor site) consistent with methods described previously. Figure 4–8 display topoplots of group level averages of signed r-squared values and coherence values and do not use flipped-maps. Therefore, the maps for the left hand movements represent “an average” of these measures from impaired hand movements (as the majority of participants in this group were left-hand impaired) and non-impaired left hand movements (minority of subjects). In the same fashion, the maps for the right hand movements represent an average of these measures from impaired hand movements (minority of participants in this group were right-hand impaired) and non-impaired right hand movements (majority of subjects). In essence, the authors didn’t flip the maps that are displayed in the figures.

### EEG Results

#### R-Squared

The signed r-squared value (at the ipsilesional C4 or C3 sites) for the Mu (8–12 Hz) rhythm significantly decreased in the post-therapy stage compared to the pre-therapy stage [one-tailed paired *t*-test: *t*(20) = 1.85; *p* = 0.039; meanPRE = -0.142; meanPOST = -0.161], while the subject attempted movements of the impaired hand (Figure 4). This suggests that as the result of the intervention sessions, the “desynchronization” of the Mu rhythm signals significantly increases post-therapy at the ipsilesional motor site. The bar graph displays the significant difference in the group mean r-squared values. The signed r-squared values of the Mu band signals decreased also post-therapy at the contralesional motor site during attempted movements of the impaired hand, but these differences did not reach significance [one-tailed paired *t*-test: *t*(20) = 1.24; *p* = 0.114; meanPRE = -0.131; meanPOST = -0.145]. Figure 5 shows topographies of group-level grand averaged r-squared values obtained from data of 21 participants. Topoplots for both Mu and Beta bands are shown. While the presented results only describe changes in the Mu band, statistics from beta band did not reach significance. The Mu band and Beta band signals were both used for BCI control.

#### LI

Laterality index (LI) values, calculated from coherence estimates at C3 and C4 sites from Beta band (18–26 Hz) signals, decreased in post-therapy stage compared to the pre-therapy stage [one-tailed paired *t*-test: *t*(20) = 0.983, *p* = 0.168; meanPRE = 0.017; meanPOST = 0.009] while the subjects attempted movements of the impaired hand, although this change did not achieve statistical significance (Figure 6). This suggests that as a result of the intervention sessions, coherence in the affected motor site compared to the contralesional site showed a statistically insignificant increase at group level. Figure 6 shows topographies of group-level grand averaged coherence values from data of 21 subjects. The value entered in each electrode site of the mentioned topographies represents the average coherence of that site with all others.

#### Imaginary Movement

Although no significant results were obtained from the analyses of data from imaginary movement trials, the topographical maps of r-squared and coherence values showed meaningful spatial distributions (Figure 7, 8). Figure 7, 8 show topographical maps (topoplots) of grand averages for signed r-squared values at group level (*n* = 21) and topoplots of grand averaged coherence values at group level for Mu (8–12 Hz) rhythm and Beta (18–26 Hz) band during imaginary movement trials, respectively. As the protocol was designed to train and reward attempted movements, it is possible participants were not sufficiently able to master imagined movement related SMR modulation.

#### Amount of Intervention: Number of Runs

**FIGURE 9:**
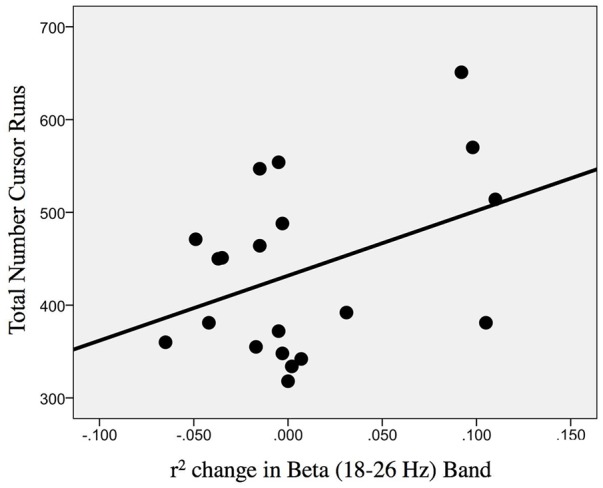
Association between the change in r-squared values (Beta band, 18–26 Hz) as the result of BCI training with the total number of cursor trial (i.e., intervention) runs [*r*(20) = 0.393; *p* = 0.043].

The change in r-squared values (Beta band) in the ipsilesional hemisphere motor site during impaired hand attempted movements, following the intervention, showed a significant correlation with the total number of cursor trials (i.e., amount of BCI practice) runs [*r*(20) = 0.393; *p* = 0.043] (Figure 9). Item number eight in Section “Description of the EEG Data Analysis” clarifies that for the calculation of change (POST–PRE) in signed r-squared values the following formula was used: -(POST–PRE), so one would again obtain positive numbers for “increases” in desynchronization. This was done for easier interpretation of the associations of r-squared changes with behavior changes as the result of EEG-BCI-FES intervention and in accord with the previously described methods. In essence, the positive correlation suggests that a greater amount of BCI practice relates to “greater” ERD.

#### Influences on Primary Outcome Measure

**FIGURE 10:**
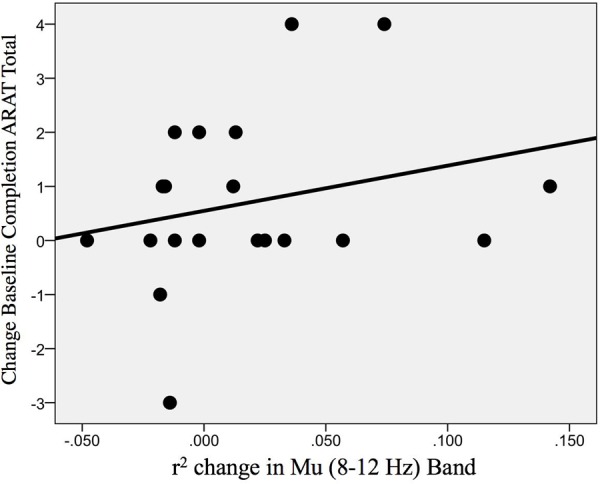
Association between the change in r-squared values (Mu rhythm, 8–12 Hz) as the result of BCI training with the change in ARAT scores (obtained post-intervention in comparison to baseline) [ρ(20) = 0.30; *p* = 0.098].

In addition, the change in r-squared values (Mu rhythm) in the ipsilesional hemisphere motor site during impaired hand attempted movements, as the result of EEG-BCI-FES intervention, showed a positive, non-statistically significant correlation with the change in ARAT scores (obtained post-therapy in comparison to baseline) [ρ(20) = 0.30; *p* = 0.098] (Figure 10).

#### Influences of Stroke and ERD on Baseline Behavioral Measures of Function and Capacity

Finally, to test some of the fundamental assumptions of the study design and BCI device (that diminished SMR desynchronization is related to the post-stroke impairment of simple motor outputs), signed Mu and Beta r-squared values for the impaired hand attempted at baseline (i.e., first intervention session) were compared to measures of behavior (SIS, ARAT, Grip Strength), and measures of stroke-related impairments to functional capacities (NIHSS, Barthel Index) at baseline (Table 3). A few measures of behavior (Grip Strength, SIS) and independence, capacity to perform ADLs (Barthel Index, NIHSS), correlated significantly in the anticipated direction (Table 3). Relevant statistical significance tests were chosen for normal and non-normal distributions, respectively. These results suggest that SMR desynchronization may represent a fundamental neuromechanical component of motor capacity as well as motor learning and, therefore, any subsequent motor recovery potential.

**Table 3:** Summary Pearson’s r and Spearman’s ρ correlates of baseline outcome measures and EEG-based signed r-squared scores (*n* = 21).

Variables	Pre-screening MU	Pre-screening BETA
Baseline SIS hand function	ρ = -0.449, *p* = 0.041	ρ = -0.408, *p* = 0.066
Baseline SIS recovery	ρ = -0.237, *p* = 0.301	ρ = -0.384, *p* = 0.085
Baseline ARAT total	ρ = -0.367, *p* = 0.102	ρ = -0.405, *p* = 0.068
Baseline Barthel Index	ρ = -0.292, *p* = 0.199	ρ = -0.573, *p* = 0.007
Baseline grip strength	r = -0.369, *p* = 0.10	r = -0.437, *p* = 0.047
Baseline NIHSS	ρ = 0.244, *p* = 0.28	ρ = 0.473, *p* = 0.03

*Pearson’s r was used for grip strength and Spearman’s ρ was used for all other variables (two-tailed tests).*

### Adverse Events

No adverse events were reported during or after participation in the research experiment.

## Discussion

### EEG Measure and Behavior Measure Fidelity

The findings that motor cortex EEG measures during attempted movements of the impaired hand (more specifically, r-squared values reflecting desynchronization levels of Mu rhythm and Beta band signals at key motor cortical sites) are positively correlated with behavioral changes and seem to offer a measurable link between electrophysiology and behavior is in line with the hypotheses set forth in this analysis. More importantly, the significant group level changes in r-squared values post-therapy compared to pre-therapy suggest an effect of the applied EEG-BCI-FES intervention protocol which may be beneficial for motor recovery, though data are currently inconclusive. As stated in Section “Amount of Intervention: Number of Runs,” the amount of BCI practice was positively correlated with Beta band ERD of the ipsilesional motor cortex. Thus, it may be possible to conceive that, following adequate amounts of training; electrophysiological measures of connectivity such as coherence may allow additional insights into the potentials and mechanisms of functional change to the neuromuscular and neuromechanical coupling of effortful motor movement.

### EEG Utility in Stroke Rehabilitation

A strength of this design and analyses for evaluation of objective physiological or functional changes as the result of the EEG-BCI-FES intervention is that the EEG-based measures extracted and compared were obtained immediately before, and immediately following each BCI intervention session (e.g., EEG-BCI-FES based rehabilitative intervention), at the pre an post screening periods (Figure 2). By comparing the EEG-based measure (i.e., r-squared, coherence) changes at post- to pre-intervention session, this allowed a more controlled evaluation of the specific effects of EEG-BCI-FES intervention. In addition, because the EEG signals are continuously recorded as part of the procedure, EEG-based measures can be obtained with no additional cost at any desired time (restricted only by the short interval required to extract reliable individual measure scores from spectral analyses of the signals). Furthermore, the study design allowed extraction and comparison of spectral estimates separately from attempted actual, as well as imaginary, hand movements. The current study did not, however, obtain statistically significant results when evaluating changes in EEG-based measures from imaginary hand movements at group level. This may be influenced by limited and insufficient time spent training participants to use imagination to properly control their SMR activity. Participants were explicitly and repeatedly instructed to attempt actual hand movements in an unblinded effort to regain or relearn volitional movement of their hands. None-the-less, reasonably distributed spatial maps of EEG activity in the SMR frequencies of interest from motor imagery attempts were observed (Figure 8). It is important, however, to note that motor imagery approaches are increasingly popular (Hatem et al., 2016; Irimia et al., 2016) and might be a relevant means of EEG-BCI translation, particularly in stroke patients with severely impaired motor function.

### Limitations

These results suggest that EEG-BCI-FES has the potential to induce neuroplastic change and aid recovery of UE paresis. However, this analysis was constrained by sample size and heterogeneity in lesion location, level of impairment, and time since stroke. Greater power is needed to adequately generalize these results. Utilizing a larger and more homogeneous subject cohort could allow for more generalizable conclusions in future research. Further, 16 electrodes were used in EEG signal data acquisition and EEG were recorded only during the intervention phase and at no other time during the study. While there is no EEG data recorded in the control period to compare with the recordings during intervention, there are brain (EEG) – behavior correlations specifically in EEG measures associated with motor function originating specifically from electrodes (C3/C4) (Figure 3) overlying regions conventionally attributed to motor function. Scalp or surface level EEG recordings are understood to read the dipolar or regional sources assumed to represent the synchronous activity of hundreds of thousands of underlying neighboring neurons. It is therefore possible that even if stroke lesions damage traditional cortical areas associated with motor output (primary motor cortex), perilesional brain regions, as well as established functional areas (pre-motor area and supplementary motor areas) may contribute to ipsilesional signal recordings sufficient to drive successful classifier activation (i.e., brain signal oscillations ‘discrete’ enough for the BCI to interpret SMR change and execute the relevant device or output command – in this case, horizontal cursor movement and facilitated FES activation) of a BCI.

#### Spatial Coverage and Sampling

It is generally understood that using 16 electrodes is insufficient for source localization, especially given the limited spatial coverage and non-equidistant spacing of the electrodes in this cap array (Figure 3) and thus, the present analysis does not consider such undertakings. In future research, the directionality and polarity of EEG-BCI-FES associated changes may lead to better understanding of the nature and sequence of motor related neuroplasticity as well as the neuroplastic influences of BCI technologies. Source reconstruction will be done once the sample size increases to sufficiently examine this aspect in a subset of stroke participants with homogeneity in lesion location. Given the heterogeneity of lesion location in the existing sample set, source localization might be premature.

##### Statistical approach

Such heterogeneity and restricted sample size similarly dissuaded the authors from attempting further conservative controls, such as multiple comparisons corrections. The authors conceived that further conservative data manipulations may wash out any potential (‘trending to’) significant relationships the authors or other groups may want to follow-up with future research. This manuscript, part of a larger on-going clinical trial, is an interim analysis which sought to elucidate any significant trends in the data as the study progressed so as to inform our future questioning of the data and to be better prepared to identify and test potentially significant interactions and factors in the larger post-stroke population.

##### Nature of the academic research environment

This is an on-going study in its seventh year of data acquisition and participant enrollment. Various project personnel have undergone and supervised the staffing, training, and data acquisition of this trial during its course. The authors work hard to best minimize differences in acquisition of study measures through extensive and repeated training of personnel.

### Future Scope

Despite the existing challenges to providing evidence-based treatment strategies in the stroke rehabilitation field, combined therapies may be used to achieve the maximal motor function recovery for participants (Oostenveld et al., 2011). Development of effective strategies for rehabilitation of impaired motor functions in stroke patients, as well as for monitoring and evaluation of changes during an applied intervention is yet needed.

## Conclusion

### EEG Conclusions

Non-invasive EEG-based measures of motor cortex function, such as r-squared (reflecting desynchronization levels of the relevant SMR rhythms), could provide an efficient means of tracking and even predicting functional changes in stroke patients during the course of the EEG-BCI-FES intervention. As ERD changes were reported at the group level, and given the heterogeneity in the sample, it may be argued that the reported changes not only suggest a change in function for the majority of participants (despite few changes attaining clinically significant differences) but also, given more selective sampling and independent variable control, an even more clinically relevant relationship between ERD and recovery may exist. Tracking SMR modulations may be a potential predictor of recovery or indicator of recovery potential in a patient.

### BCI Conclusions

The observed effects to motor measures might also be a consequence of challenging and rewarding movements associated with (ADLs), which the participants previously may have thought to be impossible or too difficult to produce successfully. BCI intervention may help challenge a survivor’s individual conception of their limitations by pushing a participant to use the affected hand and rewarding them (according to an anticipatable, clear, and consistent schedule) for doing so. This is to suggest that the minimal gains observed by most participants, in comparison to the significant gains obtained by some, and their absence in others, may be related to the encouragement of attempting previously ineffective motor behaviors. It is possible the statistically significant gains observed, supported by the higher incidence of significance in subjective measures than the number of lab-based objective measures, could be the result of the specific reward structure of the design in addition to, or more so than any reliable neuromechanical or electrophysiological contributions.

#### Biological Limitations and Contribution of Learning Theories

If normal muscle synergies (e.g., the same muscles act to abduct one’s arm each time, in a healthy adult) are disrupted by an insult such as stroke, robbing the motor circuity of its primary output components (e.g., central nervous system efference to peripheral nervous system effectors), residual functional capacities are limited by the ability of the system to retrain or re-map (link) the CNS commands to PNS effectors (Power et al., 2011). Successful BCI intervention must connect the peripheral muscle activation with the muscle effectors necessary to execute a motor function according to the user’s CNS command to do so. Unfortunately, retraining the processes of the descending motor system is not always an option as stroke often results in irreparable tissue damage or death to motor pathways and even their sensorimotor confederates. Post-stroke neuronal loss alters recruitment of downstream muscle synergies (Cheung et al., 2009), and alters a synergy’s internal structure (Roh et al., 2013) depending on stroke severity (Roh et al., 2015). One biological mechanism left to these survivors is to adapt existing synergistic capacities toward a compensatory strategy (e.g., recruitment of novel synergistic families to accomplish a familiar movement). Thus, future BCI methodologies should rely on classical conditioning and Hebbian learning theories as well as predictive modeling for developmental guides to practice. Future BCI designs may also benefit from classification of distal muscle capacities and synergistic integrities so as to better measure, represent, facilitate, or compensate for the functional consequences of the stroke disturbed CNS and PNS circuitry.

From previously published findings (Young et al., 2014a,b,c; Song et al., 2015), we can comprehend that BCIs induce neuronal changes which, in turn, might help the participants challenge their paresis or perceived disabilities (Dromerick et al., 2009; Remsik et al., 2016), as they access or develop (i.e., train) new functional aptitudes, or reinvigorate old synergies and neural networks dampened by insult (Remsik et al., 2016). Participants may have the perception that their ability has improved or changed; however, when assessed by objective measures, those perceptions, at least here, are not always confirmed at equal magnitude. The authors posit that neural changes reported by other groups and in our previous publications may not always manifest as clinically significant objective changes in motor function (Wenger et al., 2017) because there is either, or both a threshold effect, or a missing component to this type of intervention (such as sufficient dosing parameters, subject selection, etc.). This opinion is potentially fortified by these results which suggest more time in intervention is related to greater electrophysiological change. Electrophysiological changes are understood to be possible biological precursors to function network change and eventually, functional behavioral change (Gazzaniga et al., 2009). Other than the simple explanation that objective lab-based measures might not reliably capture UE impairment well in stroke survivors, perhaps, as a result of engaging with this BCI intervention, this discrepancy might also arise because participants are beginning to engage their environment with the distal musculature of the impaired hand in ways they had been previously averse (unwilling) or unable to.

#### More Intervention

Losing strategies, more often than not, do not win (e.g., adaptive vs. maladaptive behaviors). Maladaptive associations may simply need more time to be pruned away and relevant adaptive associations strengthened by increased and more highly structured reinforcement (Gazzaniga et al., 2009; Wenger et al., 2017). If one assumes such a threshold effect, the neural-remodeling realized in these participants may suggest that more intervention trials were needed to translate to clinically significant, not just relevant, changes in objective measures of function. Results suggest a relationship between more trials and greater outcome measure change, paralleling a concept associated with training, or learning a new motor skill: practice makes permanent. It may be that amount of intervention, or inadequate ‘dosage’ in this case, explains the weak translation of observed brain level changes into behavioral gains for this cohort. Little evidence has thus far been offered to suggest an optimal BCI regimen. Perhaps there is even an upper limit, or even some consequence of fatigue. It is therefore suggested that future research address these questions and aim to better understand dose-response relationships and independent variable (lesion location, lesion volume, time since stroke, comorbid impairments, etc.) contributions to predict recovery potential and more efficaciously prescribe BCI intervention as therapy. All BCI research would benefit by a concerted effort to identify a therapeutic index for various BCI interventions (regimens) as well as attempt to target ideal patient profiles for prescription of BCI intervention as a therapy.

## Author Contributions

AR and LW contributed equally to the writing of this manuscript. AR was involved in subject recruitment, staff training, data collection, data processing, data analysis, interpreting data, and writing of the manuscript. LW was involved in staff training, data collection, data processing, data analysis, interpreting data, and writing of the manuscript. KG was involved in analysis, interpreting and writing of the manuscript. KD, JT, TJ, were involved in data collection, analysis, and editing the manuscript. BY was involved in subject recruitment, staff training, data collection, data processing, data analysis, interpreting data, intellectual content, and manuscript editing. ZN was involved in subject recruitment and data collection. MW, MMc, MMa, SR, HA, RM, NT, JA, and LW were involved in data collection. VN contributed to subject recruitment, staff training, data collection, data processing, data analysis, interpreting data, manuscript editing, and intellectual content. PVK contributed to manuscript editing and intellectual content. JS was involved in recruitment of study participants, study design, and intellectual content. TK was involved in the recruitment of study participants, manuscript editing, and intellectual content. JCW, DE, and VP are co-PIs and were involved in study conception, design, staff training, manuscript editing, intellectual content, and supervised all aspects of the study.

## Conflict of Interest Statement

The authors declare that the research was conducted in the absence of any commercial or financial relationships that could be construed as a potential conflict of interest.

## Footnotes

1 https://clinicaltrials.gov/ct2/show/NCT02098265

2 https://www.mathworks.com/

## Supplementary Material

FIGURE S1 CONSORT flow diagram.

## References

[B1] Ang K. Guan C. (2013). Brain-computer interface in stroke rehabilitation. *J. Comput. Sci. Eng.* 7 139–146. 10.5626/JCSE.2013.7.2.139

[B2] Babaiasl M. Mahdioun S. H. Jaryani P. Yazdani M. (2016). A review of technological and clinical aspects of robot-aided rehabilitation of upper-extremity after stroke. *Disabil. Rehabil. Assist. Technol.* 11 263–280. 10.3109/17483107.2014.1002539 25600057

[B3] Benjamin E. J. Blaha M. J. Chiuve S. E. Cushman M. Das S. R. Deo R. (2017). Heart disease and stroke statistics—2017 update: a report from the American Heart Association. *Circulation* 135 e229–e445. 10.1161/CIR.0000000000000485 28122885 PMC5408160

[B4] Birbaumer N. Weber C. Neuper C. Buch E. Haapen K. Cohen L. (2006). Physiological regulation of thinking: brain-computer interface (BCI) research. *Prog. Brain Res.* 159 369–391. 10.1016/S0079-6123(06)59024-7 17071243

[B5] Bundy D. T. Souders L. Baranyai K. Leonard L. Schalk G. Coker R. (2017). Contralesional brain-computer interface control of a powered exoskeleton for motor recovery in chronic stroke survivors. *Stroke* 48 1908–1915. 10.1161/STROKEAHA.116.016304 28550098 PMC5482564

[B6] Caria A. Weber C. Brötz D. Ramos A. Ticini L. F. Gharabaghi A. (2011). Chronic stroke recovery after combined BCI training and physiotherapy: a case report. *Psychophysiology* 48 578–582. 10.1111/j.1469-8986.2010.01117.x 20718931

[B7] Cheung V. C. Piron L. Agostini M. Silvoni S. Turolla A. Bizzi E. (2009). Stability of muscle synergies for voluntary actions after cortical stroke in humans. *Proc. Natl. Acad. Sci. U.S.A.* 106 19563–19568. 10.1073/pnas.0910114106 19880747 PMC2780765

[B8] Daly J. J. Cheng R. Rogers J. Litinas K. Hrovat K. Dohring M. (2009). Feasibility of a new application of noninvasive Brain Computer Interface (BCI): a case study of training for recovery of volitional motor control after stroke. *J. Neurol. Phys. Ther.* 33 203–211. 10.1097/NPT.0b013e3181c1fc0b 20208465

[B9] Daly J. J. Wolpaw J. R. (2008). Brain-computer interfaces in neurological rehabilitation. *Lancet Neurol.* 7 1032–1043. 10.1016/S1474-4422(08)70223-0 18835541

[B10] Dromerick A. W. Lang C. E. Birkenmeier R. L. Wagner J. M. Miller J. P. Videen T. O. (2009). Very early constraint-induced movement during stroke rehabilitation (VECTORS): a single-center RCT. *Neurology* 73 195–201. 10.1212/WNL.0b013e3181ab2b27 19458319 PMC2715572

[B11] Felton E. A. Wilson J. A. Williams J. C. Garell P. C. (2007). Electrocorticographically controlled brain-computer interfaces using motor and sensory imagery in patients with temporary subdural electrode implants. Report of four cases. *J. Neurosurg.* 106 495–500. 10.3171/jns.2007.106.3.495 17367076

[B12] Fleet A. Page S. J. MacKay-Lyons M. Boe S. G. (2014). Modified constraint-induced movement therapy for upper extremity recovery post stroke: what is the evidence? *Top. Stroke Rehabil.* 21 319–331. 10.1310/tsr2104-319 25150664

[B13] Gazzaniga M. S. (2005). Forty-five years of split-brain research and still going strong. *Nat. Rev. Neurosci.* 6 653–659. 10.1038/nrn1723 16062172

[B14] Gazzaniga M. S. Ivry R. B. Mangun G. R. (2009). *Cognitive Neuroscience: The Biology of the Mind* 3rd Edn. New York, NY: W W NORTON.

[B15] Hatem S. M. Saussez G. Della Faille M. Prist V. Zhang X. Dispa D. (2016). Rehabilitation of motor function after stroke: a multiple systematic review focused on techniques to stimulate upper extremity recovery. *Front. Hum. Neurosci.* 10:442. 10.3389/fnhum.2016.00442 27679565 PMC5020059

[B16] Hill N. J. Lal T. N. Schröder M. Hinterberger T. Wilhelm B. Nijboer F. (2006). Classifying EEG, and ECoG signals without subject training for fast BCI implementation: comparison of nonparalyzed, and completely paralyzed subjects. *IEEE Trans. Neural Syst. Rehabil. Eng.* 14 183–186. 10.1109/TNSRE.2006.875548 16792289

[B17] Howlett O. A. Lannin N. A. Ada L. McKinstry C. (2015). Functional electrical stimulation improves activity after stroke: a systematic review with meta-analysis. *Arch. Phys. Med. Rehabil.* 96 934–943. 10.1016/j.apmr.2015.01.013 25634620

[B18] Hsieh C. L. Hsueh I. P. Chiang F. M. Lin P. H. (1998). Inter-rater reliability and validity of the action research arm test in stroke patients. *Age. Ageing* 27 107–113. 10.1093/ageing/27.2.107 16296669

[B19] Irimia D. Sabathiel N. Ortner R. Poboroniuc M. Coon W. Allison B. Z. (2016). recoveriX: a new BCI-based technology for persons with stroke. *Conf. Proc. IEEE Eng. Med. Biol. Soc.* 2016 1504–1507. 10.1109/EMBC.2016.7590995 28268612

[B20] Jones T. A. (2017). Motor compensation and its effects on neural reorganization after stroke. *Nat. Rev. Neurosci.* 18 267–280. 10.1038/nrn.2017.26 28331232 PMC6289262

[B21] Kaczmarek A. K. (2011). The tongue display unit (TDU) for electrotactile spatiotemporal pattern presentation. *Sci. Iran.* 18 1476–1485. 10.1016/j.scient.2011.08.020 28748231 PMC5523951

[B22] Kollen B. Kwakkel G. Lindeman E. (2006). Functional recovery after stroke: a review of current developments in stroke rehabilitation research. *Rev. Recent. Clin. Trials* 1 75–80. 10.2174/157488706775246111 18393783

[B23] Krafi G. H. Fitts S. S. Hammond M. C. (1992). Techniques to improve function of the arm and hand in chronic hemiplegia. *Arch. Phys. Med. Rehabil.* 73 220–227. 1543423

[B24] Kuiken T. A. Schultz Feuser A. E. Barlow A. K. (2013). *Targeted Muscle Reinnervation: A Neural Interface for Artificial Limbs*. Didcot: Taylor & Francis 10.1201/b15079

[B25] Lang C. E. Edwards D. F. Birkenmeier R. L. Dromerick A. W. (2008). Estimating minimal clinically important differences of upper extremity measures early after stroke. *Arch. Phys. Med. Rehabil.* 89 1693. 10.1016/j.apmr.2008.02.022 18760153 PMC2819021

[B26] Laver K. George S. Thomas S. Deutsch J. E. Crotty M. (2015). Virtual reality for stroke rehabilitation: an abridged version of a Cochrane review. *Eur. J. Phys. Rehabil. Med.* 51 497–506. 26158918

[B27] Leuthardt E. C. Schalk G. Wolpaw J. R. Ojemann J. G. Moran D. W. (2004). A brain-computer interface using electrocorticographic signals in humans. *J. Neural Eng.* 1 63–71. 10.1088/1741-2560/1/2/001 15876624

[B28] Lindenberg R. Renga V. Zhu L. L. Nair D. Schlaug G. (2010). Bihemispheric brain stimulation facilitates motor recovery in chronic stroke patients. *Neurology* 75 2176–2184. 10.1212/WNL.0b013e318202013a 21068427 PMC3013585

[B29] Maris E. Oostenveld R. (2007). Nonparametric statistical testing of EEG- and MEG-data. *J. Neurosci. Methods* 164 177–190. 10.1016/j.jneumeth.2007.03.024 17517438

[B30] Mathiowetz V. Weber K. Kashman N. Volland G. (1985). Adult norms for the nine-hole peg test of finger dexterity. *Occup. Ther. J. Res.* 5 24–38. 10.1177/153944928500500102 3160243

[B31] McFarland D. J. Krusienski D. J. Wolpaw J. R. (2006). Brain-computer interface signal processing at the Wadsworth Center: mu and sensorimotor beta rhythms. *Prog. Brain Res.* 159 411–419. 10.1016/S0079-6123(06)59026-0 17071245

[B32] Mitra P. P. Pesaran B. (1999). Analysis of dynamic brain imaging data. *Biophys. J.* 76 691–708. 10.1016/S0006-3495(99)77236-X 9929474 PMC1300074

[B33] Moe J. H. Post H. W. (1962). Functional electrical stimulation for ambulation in hemiplegia. *J. Lancet* 82 285–288. 14474974

[B34] Muralidharan A. Chae J. Taylor D. M. (2011). Extracting attempted hand movements from EEGs in people with complete hand paralysis following stroke. *Front. Neurosci.* 5:39. 10.3389/fnins.2001.00039 21472032 PMC3066795

[B35] Nair V. A. Young B. M. La C. Reiter P. Nadkarni T. N. Song J. (2015). Functional connectivity changes in the language network during stroke recovery. *Ann. Clin. Transl. Neurol.* 2 185–195. 10.1002/acn3.165 25750922 PMC4338958

[B36] Nam C. S. Jeon Y. Kim Y. J. Lee I. Park K. (2011). Movement imagery-related lateralization of event-related (de)synchronization (ERD/ERS): motor-imagery duration effects. *Clin. Neurophysiol.* 122 567–577. 10.1016/j.clinph.2010.08.002 20800538

[B37] Neuper C. Pfurtscheller G. (2001). Event-related dynamics of cortical rhythms: frequency-specific features and functional correlates. *Int. J. Psychophysiol.* 43 41–58. 10.1016/S0167-8760(01)00178-7 11742684

[B38] Oostenveld R. Fries P. Maris E. Schoffelen J. M. (2011). FieldTrip: open source software for advanced analysis of MEG, EEG, and invasive electrophysiological data. *Comput. Intell. Neurosci.* 2011:156869. 10.1155/2011/156869 21253357 PMC3021840

[B39] Ortiz G. A. Sacco R. L. (2008). *National Institutes of Health Stroke Scale (NIHSS)*. Hoboken, NJ: Wiley Encyclopedia of Clinical Trials 10.1002/9780471462422.eoct400

[B40] Ortner R. Irimia D. C. Scharinger J. Guger C. (2012). A motor imagery based brain-computer interface for stroke rehabilitation. *Stud. Health Technol. Inform.* 181 319–323. 22954880

[B41] Pangman V. C. Sloan J. Guse L. (2000). An examination of psychometric properties of the mini-mental state examination and the standardized mini-mental state examination: implications for clinical practice. *Appl. Nurs. Res.* 13 209–213. 10.1053/apnr.2000.9231 11078787

[B42] Pfurtscheller G. Neuper C. Andrew C. Edlinger G. (1997). Foot and hand area mu rhythms. *Int. J. Psychophysiol.* 26 121–135. 10.1016/S0167-8760(97)00760-5 9202999

[B43] Pfurtscheller G. Neuper C. Birbaumer N. (2005). “Human brain-computer interface (BCI),” *Motor Cortex in Voluntary Movements. A Distributed System for Distributed Functions* eds Riehle A. Vaadia E. (Boca Raton, FL: CRC Press) 367–401.

[B44] Pineda J. A. (2005). The functional significance of mu rhythms: translating “seeing” and “hearing” into “doing” *Brain Res. Rev.* 50 57–68. 10.1016/j.brainresrev.2005.04.005 15925412

[B45] Popovic D. B. Sinkaer T. Popovic M. B. (2009). Electrical stimulation as a means for achieving recoveryof function in stroke patients. *NeuroRehabilitation* 25 45–58. 19713618 10.3233/NRE-2009-0498

[B46] Power J. D. Cohen A. L. Nelson S. M. Wig G. S. Barnes K. A. Church J. A. (2011). Functional network organization of the human brain. *Neuron* 72 665–678. 10.1016/j.neuron.2011.09.006 22099467 PMC3222858

[B47] Remsik A. B. Dodd K. Williams L. Jr. Thoma J. Jacobson T. Allen J. D. (2018). Behavioral outcomes following brain–computer interface intervention for upper extremity rehabilitation in stroke: a randomized controlled trial. *Front. Neurosci.* 12:752 10.3389/fnins.2018.00752 PMC6235950 30467461

[B48] Remsik A. Young B. Vermilyea R. Kiekhoefer L. Abrams J. Evander Elmore S. (2016). A review of the progression and future implications of brain-computer interface therapies for restoration of distal upper extremity motor function after stroke. *Expert Rev. Med. Devices* 13 445–454. 10.1080/17434440.2016.1174572 27112213 PMC5131699

[B49] Roh J. Rymer W. Z. Beer R. F. (2015). Evidence for altered upper extremity muscle synergies in chronic stroke survivors with mild and moderate impairment. *Front. Hum. Neurosci.* 9:6. 10.3389/fnhum.2015.00006 25717296 PMC4324145

[B50] Roh J. Rymer W. Z. Perreault E. J. Yoo S. B. Beer R. F. (2013). Alterations in upper limb muscle synergy structure in chronic stroke survivors. *J. Neurophysiol.* 109 768–781. 10.1152/jn.00670.2012 23155178 PMC3567389

[B51] Schaechter J. D. (2004). Motor rehabilitation and brain plasticity after hemiparetic stroke. *Prog. Neurobiol.* 73 61–72. 10.1016/j.pneurobio.2004.04.001 15193779

[B52] Schalk G. McFarland D. J. Hinterberger T. Birbaumer N. Wolpaw J. R. (2004). BCI2000: a general-purpose brain-computer interface (BCI) system. *IEEE Trans. Biomed. Eng.* 51 1034–1043. 10.1109/TBME.2004.827072 15188875

[B53] Schalk G. Miller K. J. Anderson N. R. Wilson J. A. Smyth M. D. Ojemann J. G. (2008). Two-dimensional movement control using electrocorticographic signals in humans. *J. Neural Eng.* 5 75–84. 10.1088/1741-2560/5/1/008 18310813 PMC2744037

[B54] Shah S. Vanclay F. Cooper B. (1989). Improving the sensitivity of the Barthel Index for stroke rehabilitation. *J. Clin. Epidemiol.* 42 703–709. 10.1016/0895-4356(89)90065-6 2760661

[B55] Shinar D. Gross C. R. Price T. R. Banko M. Bolduc P. L. Robinson R. G. (1986). Screening for depression in stroke patients: the reliability and validity of the Center for Epidemiologic Studies Depression Scale. *Stroke* 17 241–245. 10.1161/01.STR.17.2.241 3961834

[B56] Smith M. C. Stinear C. M. (2016). Transcranial magnetic stimulation (TMS) in stroke: ready for clinical practice? *J. Clin. Neurosci.* 31 10–14. 10.1016/j.jocn.2016.01.034 27394378

[B57] Song J. Nair V. A. Young B. M. Walton L. M. Nigogosyan Z. Remsik A. (2015). DTI measures track and predict motor function outcomes in stroke rehabilitation utilizing BCI technology. *Front. Hum. Neurosci.* 9:195. 10.3389/fnhum.2015.00195 25964753 PMC4410488

[B58] Song J. Young B. M. Nigogosyan Z. Walton L. M. Nair V. A. Grogan S. W. (2014). Characterizing relationships of DTI, fMRI, and motor recovery in stroke rehabilitation utilizing brain-computer interface technology. *Front. Neuroeng.* 7:31. 10.3389/fneng.2014.00031 25120466 PMC4114288

[B59] Thakor N. V. (2013). Translating the brain-machine interface. *Sci. Transl. Med.* 5:210s17. 10.1126/scitranslmed.3007303 24197734

[B60] Thomson D. J. (1982). Spectrum estimation and harmonic analysis. *Proc. IEEE* 70 1055–1096. 10.1109/PROC.1982.12433

[B61] Varkuti B. Guan C. Pan Y. Phua K. S. Ang K. K. Kuah C. W. (2013). Resting state changes in functional connectivity correlate with movement recovery for BCI and robot-assisted upper-extremity training after stroke. *Neurorehabil. Neural Repair* 27 53–62. 10.1177/1545968312445910 22645108

[B62] Vellone E. Savini S. Fida R. Dickson V. V. Melkus G. D. E. Carod-Artal F. J. (2015). Psychometric evaluation of the stroke impact scale 3.0. *J. Cardiovasc. Nurs.* 30 229–241. 10.1097/JCN.0000000000000145 24695074

[B63] Wenger E. Brozzoli C. Lindenberger U. Lövdén M. (2017). Expansion and renormalization of human brain structure during skill acquisition. *Trends Cogn. Sci.* 21 930–939. 10.1016/j.tics.2017.09.008 29149999 PMC5697733

[B64] Wilson J. A. Walton L. M. Tyler M. Williams J. (2012). Lingual electrotactile stimulation as an alternative sensory feedback pathway for brain-computer interface applications. *J. Neural Eng.* 9:045007. 10.1088/1741-2560/9/4/045007 22832032

[B65] Wolpaw J. R. McFarland D. J. Neat G. W. Forneris C. A. (1991). An EEG-based brain-computer interface for cursor control. *Electroencephalogr. Clin. Neurophysiol.* 78 252–259. 10.1016/0013-4694(91)90040-B 1707798

[B66] Yang Q. Tong X. Schieb L. Vaughan A. S. Gillespie C. D. Wiltz J. L. (2017). Vital signs: recent trends in stroke death rates – United States, 2000-2015. *MMWR* 2017 933–939. 10.15585/mmwr.mm6635e1 28880858 PMC5689041

[B67] Young B. M. Nigogosyan Z. Nair V. A. Walton L. M. Song J. Tyler M. E. (2014a). Case report: post-stroke interventional BCI rehabilitation in an individual with preexisting sensorineural disability. *Front. Neuroeng.* 7:18. 10.3389/fneng.2014.00018 25009491 PMC4067954

[B68] Young B. M. Nigogosyan Z. Walton L. M. Song J. Nair V. A. Grogan S. W. (2014b). Changes in functional brain organization and behavioral correlations after rehabilitative therapy using a brain-computer interface. *Front. Neuroeng.* 7:26. 10.3389/fneng.2014.00026 25076886 PMC4097124

[B69] Young B. M. Williams J. Prabhakaran V. (2014c). BCI-FES: could a new rehabilitation device hold fresh promise for stroke patients? *Expert Rev. Med. Devices* 11 537–539. 10.1586/17434440.2014.941811 25060658 PMC4194138

