# Supplementary material for: Ipsilesional Mu Rhythm Desynchronization and Changes in Motor Behavior Following Post Stroke BCI Intervention for Motor Rehabilitation
Source: Front Neurosci. 2019 Mar 6;13:53. doi: 10.3389/fnins.2019.00053 (PMC6417367; doi:10.3389/fnins.2019.00053)
Supplement: FIGURE S1 — CONSORT flow diagram. [file Data_Sheet_1.doc]

**
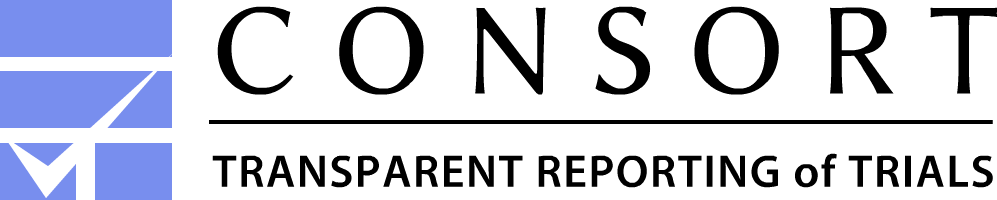
**

**CONSORT Behavioral Flow Diagram**

**Allocation**

**Analysis**

**Follow-Up**

**Enrollment**

Assessed for eligibility (n=148)

Excluded (n=111)

♦  Not meeting inclusion criteria (n=44)

♦  Travel too far for subject (n=16)

♦  Other reasons declined participation (n=14)

Analysed (n=12)
♦ Excluded from analysis (Prior to standardization of acquisition protocol (pilot data), n=3)

Discontinued intervention (broke hip, lost interest, Intervention schedule was too time consuming n=1,3,1)

Allocated to delayed Intervention (n=21)

♦ Received allocated intervention (n=19)

♦ Did not receive allocated intervention (transportation issue, n=1)

Discontinued intervention (transportation issue, passed away n=1,1)

Allocated to immediate intervention (n=16)

♦ Received allocated intervention (n=14)

♦ Did not receive allocated intervention (transportation issue, n=1)

Analysed (n=9)
♦ Excluded from analysis (Prior to standardization of acquisition protocol (pilot data), n=4)

Randomized (n=37)
